# Evaluation of Selected CYP51A1 Polymorphisms in View of Interactions with Substrate and Redox Partner

**DOI:** 10.3389/fphar.2017.00417

**Published:** 2017-06-30

**Authors:** Tadeja Režen, Iza Ogris, Marko Sever, Franci Merzel, Simona Golic Grdadolnik, Damjana Rozman

**Affiliations:** ^1^Faculty of Medicine, Centre for Functional Genomics and Bio-Chips, Institute of Biochemistry, University of LjubljanaLjubljana, Slovenia; ^2^Department of Biomolecular Structure, National Institute of ChemistryLjubljana, Slovenia

**Keywords:** CYP51A1, SNP, polymorphism, molecular dynamics simulations, POR

## Abstract

Cholesterol is essential for development, growth, and maintenance of organisms. Mutations in cholesterol biosynthetic genes are embryonic lethal and few polymorphisms have been so far associated with pathologies in humans. Previous analyses show that lanosterol 14α-demethylase (CYP51A1) from the late part of cholesterol biosynthesis has only a few missense mutations with low minor allele frequencies and low association with pathologies in humans. The aim of this study is to evaluate the role of amino acid changes in the natural missense mutations of the hCYP51A1 protein. We searched SNP databases for existing polymorphisms of *CYP51A1* and evaluated their effect on protein function. We found rare variants causing detrimental missense mutations of CYP51A1. Some missense variants were also associated with a phenotype in humans. Two missense variants have been prepared for testing enzymatic activity *in vitro* but failed to produce a P450 spectrum. We performed molecular modeling of three selected missense variants to evaluate the effect of the amino acid substitution on potential interaction with its substrate and the obligatory redox partner POR. We show that two of the variants, R277L and especially D152G, have possibly lower binding potential toward obligatory redox partner POR. D152G and R431H have also potentially lower affinity toward the substrate lanosterol. We evaluated the potential effect of damaging variants also using data from other *in vitro* CYP51A1 mutants. In conclusion, we propose to include damaging *CYP51A1* variants into personalized diagnostics to improve genetic counseling for certain rare disease phenotypes.

## Introduction

CYP51A1, lanosterol 14α-demethylase, is a cytochrome P450 involved in cholesterol biosynthesis and is present in all biological kingdoms. It was proposed to be one of the oldest cytochromes P450 and is evolutionary highly conserved. Amino acid sequence identity is 95% among mammals and about 23–34% among biological kingdoms (Lepesheva and Waterman, 2007). CYP51A1 is catalytically strict and all forms catalyze oxidative removal of the 14α-group from the sterol intermediates. The reaction occurs in three steps and requires a NADPH type reducing agent and the presence of POR (cytochrome P450 oxidoreductase). Lanosterol and 24,25-dihydrolanosterol are CYP51A1 substrates in mammals. Since the reaction catalyzed by CYP51A1 is substrate specific, there are certain amino acids conserved through biological kingdoms (Lepesheva and Waterman, 2007). These are clustered in six substrate recognition sites (SRS1-6), which are CYP51A1 specific. There are also conserved signature structures of the CYP450 superfamily, such as surroundings of heme-Cys pocket and helices B, F, G, and I (Lepesheva et al., 2003).

Cholesterol biosynthesis is an essential housekeeping pathway and knockouts of genes involved in this pathway are embryonic lethal except for a few enzymes toward the end of the synthesis pathway (Horvat et al., 2011). Mouse *Cyp51a1* knockout is lethal at day 15 *post coitum* (Keber et al., 2011). This suggests that also in humans two mutated *CYP51A1* alleles rendering no active protein would be embryonic lethal. Heterozygous knockout *Cyp51a1*^+/−^ mice are developmentally and morphologically normal and fertile; however, when challenged with high lipid diet, the response revealed a hidden susceptibility to detrimental effects of the diet (Lewinska et al., 2014). Studies in mice also revealed gender biased consequences of mutations in *Cyp51a1* (Lorbek et al., 2013; Lewinska et al., 2014; Urlep et al., 2017). Consequences of *CYP51A1* heterozygosity in humans need yet to be studied.

We have searched all available GWAS and other databases connecting SNPs with phenotype in humans. Only few phenotypes were so far associated with *CYP51A1* polymorphisms (Table 1). Several are associated with a rare variant rs2229188 causing a change of Val19 to Ala. This SNP was found associated with HDL-C level, hypertension, and lifespan (Charlesworth et al., 2009; Han et al., 2013; Yashin et al., 2015). This missense mutation lies at the N-terminal part of the protein and is responsible for interaction with the membrane; therefore, it is not directly involved in enzymatic activity (Pikuleva and Waterman, 2013). Also, Polyphen-2 or SIFT do not predict a detrimental effect of this variant. It is thus unclear how this missense variant affects the observed phenotypes. *CYP51A1* mutations were also associated with the incidence of pediatric cataracts. A mutation of Arg277 to Cys resulted in neurologically and systemically normal children with pediatric cataract (Aldahmesh et al., 2012; Khan et al., 2015). However, three other rare variants were clearly pathogenic in causing not only cataract but global developmental delay and hepatic failure (Gillespie et al., 2014, 2016; Patel et al., 2016). These are termination of protein at Trp421, change of Ile312 to Thr and Leu232 to Pro. All these mutations were labeled as damaging by Polyphen-2 or SIFT. Additionally, common *CYP51A1* SNPs in noncoding regions were associated with spontaneous premature labor, and lower LDL-C and TC in second trimester of pregnancy (Lewinska et al., 2013). Another common variant in 3′UTR region was reported to be associated with HbA1c and expression of genes in pancreas (Ren et al., 2016).

**Table 1:** *CYP51A1* polymorphisms associated with phenotypes in humans and population frequency from 1,000 genomes.

**cDNA NM_000786.3**	**Protein NP_000777.1**	**Phenotype**	**References**	**SNP ID (population frequency)**
c.1263G>A	p.W421Ter	Infantile onset central and lamellar cataract, developmental delay, brain white-matter abnormality, cryptogenic neonatal liver cirrhosis, spastic diplegia, increased lanosterol	Gillespie et al., 2014, 2016	rs141654764 (0.00001647) /
c.935T>C	p.I312T			
c.829C>T	p.R277C	Congenital cataract, neurologically and systemically normal	Aldahmesh et al., 2012; Khan et al., 2015	rs944015648 (NA)
c.695T>C	p.L232P (SRS2)	Congenital cataract, neonatal fulminant hepatic, failure, and global developmental delay	Patel et al., 2016	/
c.56T>C	p.V19A	Significant association with HDL-C level	Charlesworth et al., 2009	rs2229188 (0.00000825)
c.56T>C	p.V19A	Significant association with hypertension	Han et al., 2013	rs2229188 (0.00000825)
c.56T>C	p.V19A	Negative association with lifespan	Yashin et al., 2015	rs2229188 (0.00000825)
c.56T>C	p.V19A	Association with hypertension	Wang and Lin, 2014	rs2229188 (0.00000825)
c.595+66A>G	/	Association with spontaneous premature labor	Lewinska et al., 2013	rs57218044 (0.0363)
c.1359T>C				
				rs7797834 (0.3596)
				rs7793861 (0.3644)
c.^*^251G>C				
				rs6465348 (0.3576)
c.^*^377T>C				
				rs12673910 (0.1697)
c.^*^1016C>T				
c.^*^377T>C	/	Association with lower LDL-C and TC in second trimester	Lewinska et al., 2013	rs6465348 (0.3576)
c.251G>C	3′UTR	Associated with glycemic HbA1c	Ren et al., 2016	rs7793861 (0.3644)
		Association with expression of genes in pancreas		

*NA, non-available*.

The aim of this study was to analyse natural *CYP51A1* missense SNPs *in vitro* and by molecular dynamics modeling in relation to the substrate lanosterol and obligatory redox partner POR (cytochrome P450 oxidoreductase). We selected three missense SNPs for *in silico* prediction and two for *in vitro* testing of the effect of the polymorphism on enzymatic activity. All these amino acids are highly conserved and were identified by SIFT and Polyphen-2 as damaging. We also searched the conserved regions of the protein for missense SNPs and evaluated their effect on the enzymatic activity. We show that up to date, there are only few potentially damaging SNPs of *CYP51A1* present in the human population. Although, we could not produce an active protein with the two SNPs, we *in silico* showed a potential effect of D152G and R431H mutation on enzymatic activity by increased distance between the heme and lanosterol and a less favorable binding of lanosterol in comparison to the wild type. Calculations also predicted D152G and R277L variants to have less favorable binding to the redox partner POR.

## Materials and methods

### Search of human *CYP51A1* polymorphisms and associated phenotypes

We have searched different GWAS databases for *CYP51A1* SNPs associated with any kind of phenotype in humans. We used NHGRI-EBI GWAS Catalog (Welter et al., 2014), GRASP, and FSNP etc. We also searched all available literature by PubMed. Results of this search are shown in Table 1. We also searched and evaluated *CYP51A1* SNPs using databases: dbSNP (https://www.ncbi.nlm.nih.gov/SNP/); COSMIC (Catalog of somatic mutations in cancer) (Forbes et al., 2016); and Exome Variant Server (http://evs.gs.washington.edu/EVS/, NHLBI GO Exome Sequencing Project). We used PolyPhen-2 and SIFT for prediction of variant effect (Ng and Henikoff, 2003; Adzhubei et al., 2010). We used NP_000777.1 as a reference for designating amino acid position.

### Preparation of mutant proteins

Two mutant CYP51A1 proteins were prepared by site-directed mutagenesis of wild type human *CYP51A1* cloned in pCWori+ plasmid. The mutants were Arg431His and Arg277Leu. Site-directed mutagenesis was performed using QuikChange™ Site-Directed Mutagenesis kit (Stratagene, La Jolla, CA, USA) and primers R277L (forward primer: 5′-GCAATCCAGAAACTCAGACAGTCTCAAG-3′ and reverse primer: 5′-CTTGAGACTGTCTGAGTTTCTGGATTGC-3′), R431H (forward primer: 5′-GGACTTTAATCCTGATCACTACTTACAGGATAACC-3′ and reverse primer: 5′-GGTTATCCTGTAAGTAGTGATCAGGATTAAAGTCC-3′) (Sigma-Aldrich, Munich, Germany) according to manufacturer recommendations with some changes in PCR run (longer elongation time and higher concentration of primers). Plasmids were transformed in the *E. coli* strain HMS174 (DE3) (Novagen, Darmstadt, Germany) and isolated using GenElute HP Plasmid Miniprep Kit (Sigma Aldrich, Munich, Germany). Plasmids were linearized using HindIII (Roche, Basel, Switzerland) and size was checked using 0.7% agarose gel. Successful change in code and the *CYP51A1* insert in the plasmid was confirmed by sequencing. Expression was carried out at 26°C after induction with IPTG (Isopropyl β-D-1-thiogalactopyranoside) and addition of δ-aminolevulinic acid for 45 h. The cells were pelleted and resuspended in 50 mM potassium phosphate, pH 7.4 (containing 10% glycerol, 0.1% Triton X-100, 200 mM NaCl, 0.5 mM phenylmethylsulfonyl fluoride, and protease inhibitor cocktail). After sonification on ice for 4 × 15 s, Triton X-100 was added to final 0.4% concentration followed with ultracentrifugation at 100,000 × g for 1 h. Proteins were purified with Ni-nitriloacetic-acid agarose column (Qiagen, Valencia, CA, USA) as described before (Lepesheva et al., 2003). Concentration and reduced CO difference spectra was determined as described before (Zelenko et al., 2014). We used 200 μl of 3 μM protein extract for measurement of CO spectrum. Western blot analysis was done as described before (Lorbek et al., 2015).

### Molecular dynamics simulations (MD) of CYP51A1 mutants

In addition to the wild type structure of CYP51A1 protein, we modeled its three mutants: Asp152Gly, Arg431His, and Arg277Leu, in two different environments, aqueous solution and in complex with cytochrome P450 reductase (POR). For CYP51A1 we have chosen 3LD6 structure and for POR 3ES9 structure from RCSB Protein Data Bank (Berman et al., 2000). The point mutations were prepared using “residue mutagenesis” procedure in Pymol [2]. Appropriate rotamers were chosen, taking into account neighboring amino acids, as not to significantly perturb the structure of the protein. Following our previous work (Lewinska et al., 2013) we have generated appropriate structural models of CYP51A1-heme-lanosterol complexes and performed all-atom molecular dynamics (MD) simulations of all systems using the software package CHARMM (Brooks et al., 1983) and the available force field (MacKerell et al., 1998). The force-field parameterization for lanosterol was taken from work of Cournia et al. (2005). In addition, the proper connection between the heme (Fe) and Cys449 (S) atom was achieved by the correct protonation state of Cys449 and an additional harmonic restraint. Each protein was embedded in a tetragonal simulation cell with dimensions of 75 × 80 × 80 Å in explicit water environment modeled by the TIP3P water model (Jorgensen et al., 1983). Initially we performed 100 steps of steepest descent minimization followed by 500 steps of adopted-basis Newton-Raphson minimization. Subsequent MD simulations were run at constant pressure of 1 bar and temperature 300 K with a time-step of 1 fs for 10 ns to enable thermodynamic equilibration of the each system.

Protein structures obtained at the end of each all-atom simulation was taken further as the starting structures for docking of proteins. Docking was achieved using the server-side docking software Zdock (Pierce et al., 2014). According to the work (Sündermann and Oostenbrink, 2013) the POR should occupy an elongated conformation when forming contact with CYP51A1. Therefore, we used weak harmonic constraints on extended-parts of the POR through CHARMM module MMFP GEO to smoothly stretch the protein to a structure resembling the one shown in the reference (Sündermann and Oostenbrink, 2013) during short term MD simulations. While for the CYP51A1 it is not known explicitly which residues take part in the interaction with POR, such information is available for other members in the CYP family, namely for CYP2D6 and CYPB24. In addition the similarity of electrostatic surfaces between proteins of the CYP family can be used as an indication of the interaction surface. We performed a sequence alignment followed by a structural superposition with refinement of different CYP proteins in Pymol. This method is known to be reliable if sequence homology is around 30%. Our proteins have 26–28% sequence identity based on the output of the BLAST algorithm, thus satisfying this criterion (Altschul et al., 1990). In addition we also found favorable electrostatic complementarity of protein contact surfaces.

Based on clustering of residues in the wild type and mutants around key interacting residues of CYP2D6 and CYPB24, we choose the following residues as key interacting residues in CYP51A1: K127, A172, K175, K364, K442, R452, N459, and corresponding residues of POR: E92, 93, 142. The isolated protein structures were subsequently docked using an online protein-protein docking software Zdock (Pierce et al., 2014) producing between 2 and 10 different poses of the complex. The three poses with the best result regarding the scoring function were taken for further evaluation. Based on the position of key homologous interacting residues with known favorable interactions for between CYP and POR the best-scored pose was chosen. For the final production run of the docked complexes, we used the implicit solvent method FACTS (Haberthür and Caflisch, 2008) to perform a more efficient sampling of the conformational space. The reliability of the FACTS method for the present system, was demonstrated by high level agreement between averages over FACTS and explicit solvent trajectories. FACTS parameters were set as follows: Tfps 3, dielectric constant 1.0, and gamma constant 0.015. The MD simulations were run at a temperature of 300 K with a time step of 1 fs. The non-bonded interactions were treated using a set of cut-offs. The distance cut off in generating the list of pairs was set to 20 Å. At 16 Å the switching function eliminated all contributions to the overall energy from pairwise interactions. At 14 Å the smoothing function began to reduce a pair's contribution. Last 20 ns of overall 40 ns of performed FACTS MD simulations were used for analysis. Simulated systems were visualized using Pymol. Analysis of results was done by in-house developed tools in addition to those available in CHARMM.

## Results

### *CYP51A1* variants in human population

We searched the databases for SNPs in the human population causing missense mutations in any of the known regions important for substrate recognition, enzymatic activity, POR interaction and azole binding (Nitahara et al., 2001; Lepesheva et al., 2003; Strushkevich et al., 2010). We also compared existing SNPs to previously published *in vitro* mutations of human and rat *CYP51A1* and proposed a potential effect of such mutation in humans (Table 2). For some variants we can conclude that they probably affect enzymatic activity of CYP51A1, for example Y137C, D152G, Y233^*^, and H320P, while for other variants *in vitro* mutants do not confirm any effect on enzymatic activity.

**Table 2:** Human SNP and rat/human *in vitro* mutant pairs.

**Human SNP**	**Rat/human mutant**	**Mutant effect**	**Predicted human SNP effect**
Y137C	ratY131F/S,	No protein, no activity	No activity
	humanY137F	55% expr, no activity, spectr. ok	
	humanY137A	Decrease in binding of substrate	
R139H	ratR133G	Normal activity	Unknown
D152G/N	ratD146A	Normal express, 106% activity	Decreased activity
	humanD152A	70% expression, 54% activity, 4 times decrease in turnover number	
Y233^*^	ratY227F	55% activity	Lower activity
H242R	humanH242A	Destabilization of the protein	Unknown effect
H320P	ratH314F/A/K/D	Lower activity (42.6, 34.9, 20.2, 14)	Lower activity of the enzyme
	humanH320A	Destabilization of protein, higher affinity for products	
T325A	ratT319A	Normal activity	Normal activity
R383V/L	humanR383A	Decrease in binding of substrate	Unknown
R388Ter	ratR382A	No protein	No protein
T492A	ratT486A	Normal activity	Normal activity
T496I	ratT490A	Normal activity	Normal activity
E375^*^	ratE369A	No protein	No protein

*Rat and humans in vitro mutants were published previously (Nitahara et al., 2001; Lepesheva et al., 2003; Bellamine et al., 2004; Mukha et al., 2011)*.

Search of the dbSNP database revealed less than 200 SNPs causing a missense mutation in *CYP51A1*. Majority are labeled benign or tolerated by Polyphen-2 or SIFT. Looking at the regions not previously connected to any aspects of the protein activity we found 12 rare variants predicted deleterious and damaging by both softwares (Table 3). Next, we searched only for SNPs existing in SRS regions or any other region predicted to be involved in any aspect of enzymatic activity (Table 4). In total, we found 33 missense mutations. In SRS1 there are 8 missense mutations and majority of them were predicted damaging by SIFT and Polyphen-2. Both variants in SRS2 region are pathogenic. One was found only once in carcinoma tissue (Y233^*^), and second (L232P) in only one family causing congenital cataract, neonatal hepatic failure and global developmental delay. In SRS3 three variants of the same amino acid exists with different predicted effect. In SRS4 there are only two variants with predicted damaging effect on the protein. SRS5 has many variants, but majority are predicted as tolerated or benign as nonpolar amino acids are replaced by other nonpolar amino acids. SRS6 has also only two SNPs. Based on clustering of residues in the wild type and mutants around key interacting residues of CYP2D6 and CYPB24, the following residues were chosen as POR key interacting residues in CYP51A1: K127, A172, K175, K364, K442, R452, and N459. Among these only three SNPs at two positions exist in human population and are labeled deleterious by SIFT and benign by Polyphen-2. Additional conserved regions were also searched; however, only few have missense mutations with potentially deleterious effect existing in human population. Very interesting are also variants at positions predicted to interact with azoles (Strushkevich et al., 2010). There are two variants where large aromatic tryptophan is changed to a small polar serine. This change could potentially affect binding affinity toward azoles.

**Table 3:** *CYP51A1* SNPs predicted as deleterious or damaging by SIFT and PolyPhen-2 in regions not connected to enzymatic activity.

**dbSNP**	**SIFT (score)**	**PolyPhen-2 (score)**	**SNP position**	**Amino-acid location**
rs372875744	Damaging (0.03)	Probably damaging (0.993)	N125H	Helix B
rs535433995	Damaging (0.01)	Probably damaging (0.975)	H177R	Helix D
rs151249652	Damaging (0.01)	Probably damaging (0.984)	E194C	Beta sheet 3-1
rs536125410	Damaging (0)	Probably damaging (0.982)	L253S	Loop between helix F″/G, surface
rs141009880	Damaging (0.01)	Possibly damaging (0.892)	I274T	Helix G
**rs140702410**	Damaging (0)	Probably damaging (0.969)	**R277L**	Helix G
rs140118347	Damaging (0.02)	Possibly damaging (0.799)	A334S	Helix I
rs554366054	Damaging (0.02)	Probably damaging (1)	L417R	Loop between helix K′ and meander, surface
**rs138109473**	Damaging (0)	Probably damaging (0.974)	**R431H**	Meander ηk
rs542915180	Damaging (0)	Probably damaging (0.999)	R454H	Cys pocket
rs563098505	Damaging (0)	Probably damaging (0.98)	Y462D	Helix L, next to cys pocket
rs553164028	damaging (0)	Probably damaging (0.997)	R507K	C-terminal of the protein

*Bold are SNP used for molecular dynamical modeling and in vitro protein expression. Amino acid location according to Lepesheva et al. (2003)*.

**Table 4:** Reported missense SNPs in *CYP51A1* conserved regions (source: exome variant server, cosmic, dbSNP).

**SNP ID**	**Protein**	**PolyPhen-2/SIFT**	**Location**
rs368261783	Y137C	Possibly damaging (0.497)/damaging (0)	SRS1
rs750743669	S138N	Benign (0.262)/ damaging (0.04)	SRS1
rs758553106	R139C	Probably damaging (0.946)/damaging (0.03)	SRS1
rs140356336	R139H	Benign (0.029)/tolerated (0.65)	SRS1
COSM5500428	V144A	Probably damaging (0.994)/tolerated (0.29)	SRS1
rs312262912	Y151D	Probably damaging (0.962)/damaging (0)	SRS1
rs371492794	**D152G**	Probably damaging (0.988)/damaging (0.01)	SRS1
COSM1202901 rs755026542	D152N	Probably damaging (0.985)/damaging (0.01)	SRS1
rs776271983	A172V	Benign (0.101) /tolerated (0.22)	POR interaction
/	L232P	Probably damaging (0.995)/damaging (0)	SRS2
COSM353554	Y233^*^	Unknown	SRS2
COSM2863985	W245S	Probably damaging (0.981)/damaging (0.01)	Azole interaction
rs753673809	W245Ter	No protein	Azole interaction
COSM1579448	W250S	Probably damaging (0.999)/damaging (0.02)	Azole interaction
rs200921006 COSM3663398	R258C	Probably damaging (0.999)/ damaging (0)	SRS3
rs765961879 COSM1202902	R258H	Benign (0.93)/ damaging (0)	SRS3
rs765961879	R258L	Benign (0.168)/ damaging (0.01)	SRS3
rs745413412	H320P	Possibly damaging (0.899)/ damaging (0)	SRS4
rs377725460	T325A	Benign (0.359)/ damaging (0.01)	SRS4
rs760669078	P381T	Probably damaging (0.990)/tolerated (0.08)	SRS5
rs150090274	I383V	Benign (0.024)/damaging (0.05)	SRS5
rs150090274	I383L	Benign (0.143)/tolerated (0.14)	SRS5
COSM3698597	M384I	Probably damaging (0.930)/tolerated (0.34)	SRS5
rs759341868	I385F	Benign (0.075)/damaging (0.01)	SRS5
rs773893086	M386V	Benign (0.376)/tolerated (0.12)	SRS5
rs532896478	M386I	Benign (0.044)/tolerated (1)	SRS5
rs765119371	M387I	Possibly damaging (0.758)/damaging (0.05)	SRS5
COSM5986705	M387delM	Unknown	SRS5
rs748782320	R388Ter	No protein	SRS5
rs528934873	R452H	Benign (0.082)/ damaging (0.04)	POR interaction
rs779786966	R452C	Benign (0.166)/ damaging (0)	
rs768113032	T492A	Benign (0.117)/tolerated (0.15)	SRS6
rs749633381	M493V	Possibly damaging (0.545)/damaging (0.02)	SRS6

*Bold is SNP used for molecular dynamical modeling. SRS, substrate recognition site*.

Next, we selected three variants for further analyses. All three were predicted as deleterious or damaging by Polyphen-2 and SIFT (Tables 3, 4, bold). First variant, D512G lies in SRS1 region, important for enzymatic activity, where a change of small negative aspartate to small non-polar glycine is potentially pathogenic. Second variant, R431H is in meander region with unknown function. A change from positively charged aliphatic arginine to positively charged aromatic histidine has an unknown effect on enzymatic activity. Third variant, R277L lies in SRS3 region and changes large positive arginine to small non-polar leucine. As a SNP at this position was associated with pediatric cataracts, we can postulate that the mutation affects the enzymatic activity of the protein.

### Molecular dynamics simulations of natural SNPs

Molecular simulations were done simulating wild type and three natural variants present in human population (Table 3, bold). Lanosterol has apart from a single hydroxyl group mainly a nonpolar surface and therefore, interacts through hydrophobic interactions with nonpolar residues of the CYP51A1. In order to quantify the position of lanosterol in each of the simulated proteins (mutants and wild type) we have determined all CYP51A1 residues taking part in hydrogen bonding with lanosterol hydroxyl group during a production run. Hydrogen bonds (HB) were determined according to the distance criterion for donor-acceptor atoms that fit the cut-off of 2.4 Å. The relative occupation times for hydrogen bonds (HB) formed mainly with the backbone amide groups of individual residues are shown in Table 5. While the lanosterol HB distribution of the mutant R277L resembles that found for the native protein, distributions of R431H and D152G indicate clear deviation from the relative position the lanosterol has in the native protein.

**Table 5:** Relative occupation times of lanosterol's hydroxyl group and its hydrogen bond formed with a given residue (amide group of the protein backbone) during production run (20 ns).

**CYP51A1**	**Amino acid**	**τ_HB_/τ_0_ [%]**
WT	M384	24.5
	I385	65.9
	I383	1.5
R277L	M384	12.7
	I385	30.1
	I383	0.8
R431H	M493	6.3
	I494	1.2
D152G	W245	10.8
	M493	1.6
	F240	0.8

*Comparison between native CYP51A1 and its three mutants*.

**Figure 1:**
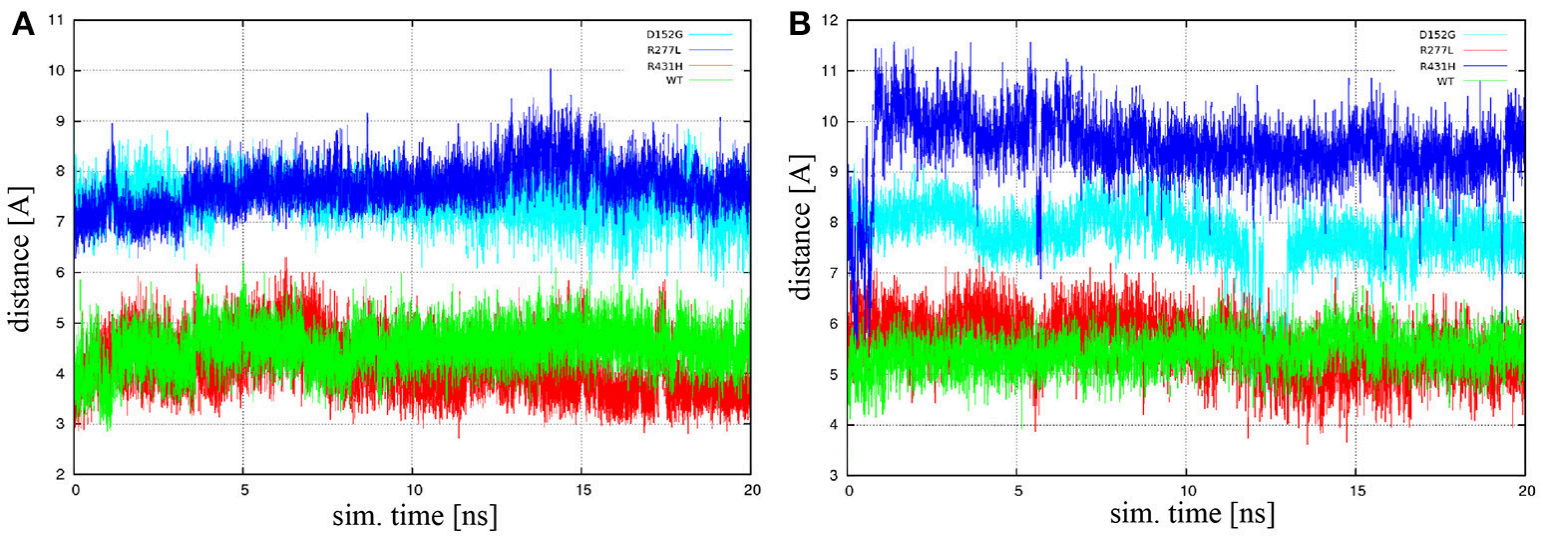
The heme iron-C30 distances as a function of simulation time (sim. time) for isolated CYP51A1 and its mutants in **(A)** aqueous solution and **(B)** complex with POR. Green line is wild type hCYP51A1, dark blue is variant R431H, red is R277L, and light blue is D152G.

Another measure for the lanosterol position is the distance between the heme iron atom and the methyl carbon C30 of lanosterol. This distance is important because lanosterol is axially coordinated to the heme iron with the methyl carbon C30, which is the lanosterol's center of oxidation. Distances between the aforementioned atoms were calculated as a function of simulation time across entire trajectories. It was found that the characteristic distance between the heme's iron and lanosterol's C30 atoms of the R277L mutant most closely resembles the wild type, while the R431H and D152G mutants distance are on average around 4 Å further away. The distances were also found to be quite stable during the production run of the simulations. The iron-C30 distances are plotted in Figure 1A for isolated CYP51A1 and its mutants in aqueous solution, while Figure 1B shows the situation for the CYP51A1 proteins in also contact with POR.

**Figure 2:**
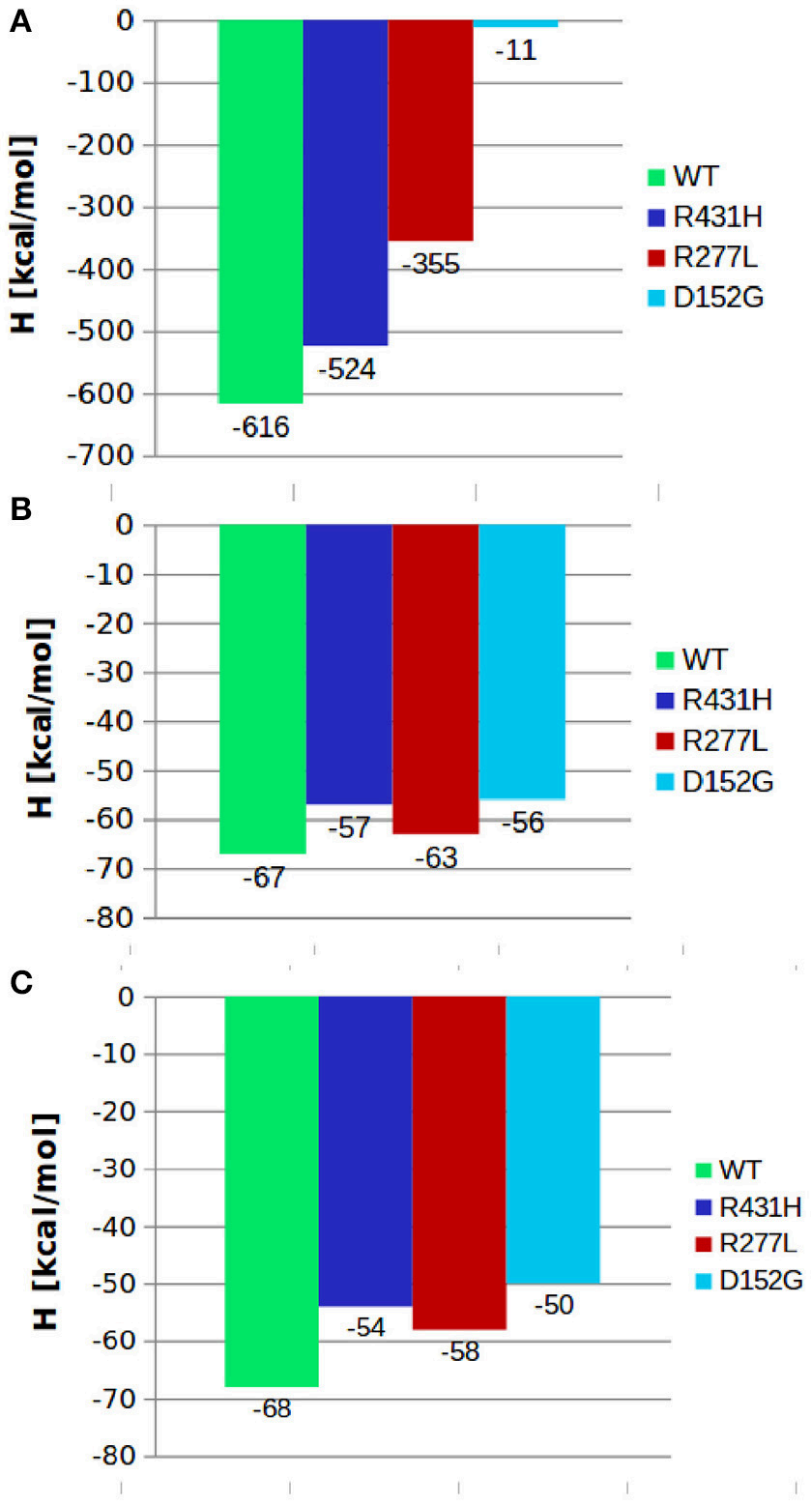
Comparison between the binding enthalpies of various mutants and the wild type CYP51A1 and **(A)** POR; **(B)** lanosterol; **(C)** lanosterol in complex with POR.

**Figure 3:**
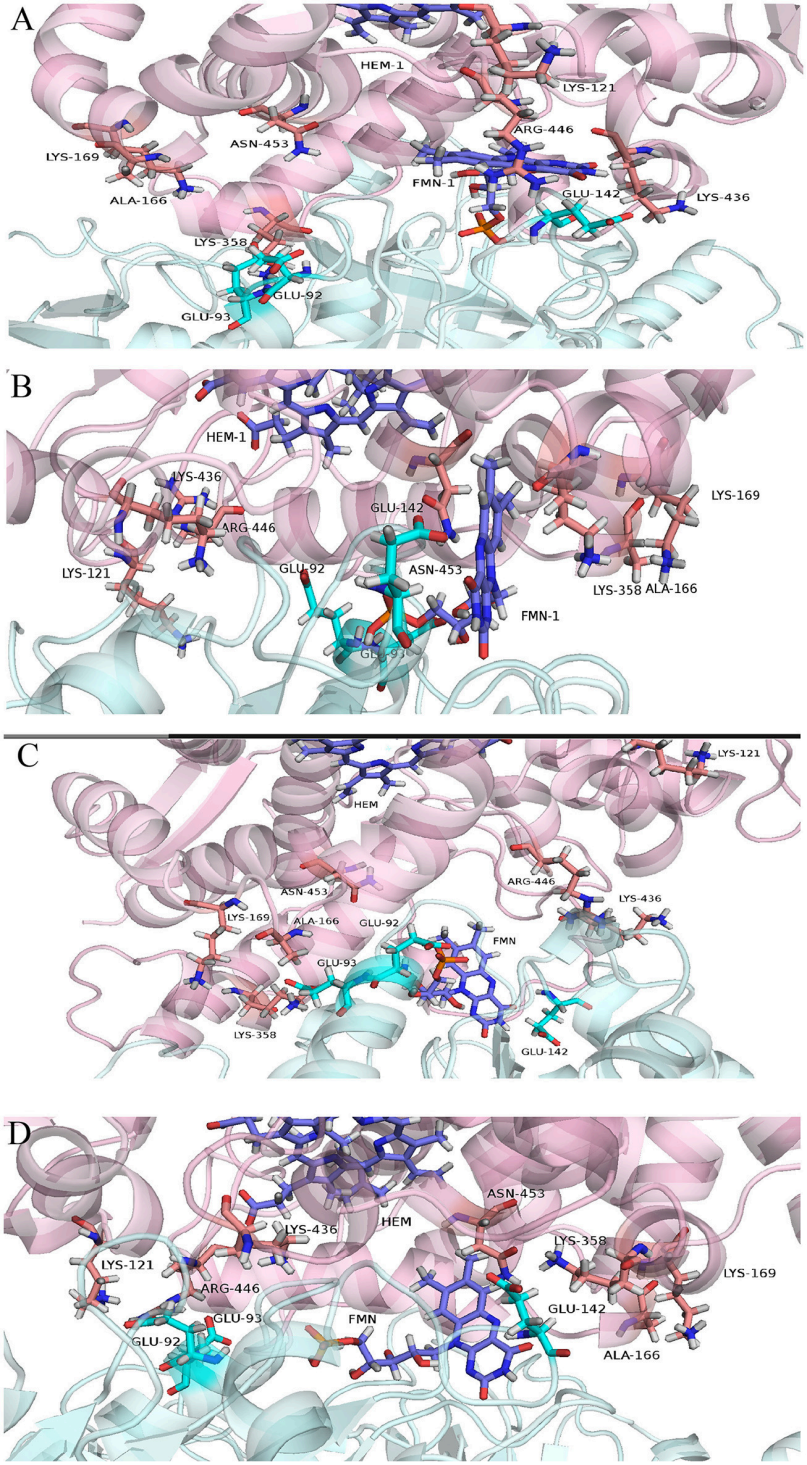
Cartoon representing predicted CYP51A1 residues interacting with POR (depicted in cyan color) in **(A)** wild type, **(B)** R277L variant, **(C)** R431H variant, and **(D)** D152G variant. (**A–D** are depicted in pink color).

Next, we calculated binding energies of individual complex by subtracting the thermodynamic averages of internal energies of individual components from the complex:

(1) $$\begin{array}{r} E_{b}=\langle U_{cmplx}\rangle -\langle U_{m1}\rangle -\langle U_{m2}\rangle , \end{array}$$

where 〈 〉 stands for the average over the entire trajectory of individual component. Each term in the above formula is calculated in a separate simulation. Here, 〈*U*_*m*1_〉 was the average internal energy of isolated lanosterol molecule in aqueous environment, 〈*U*_*m*2_〉 average internal energy of CYP protein without lanosterol and 〈*U*_*cmplx*_〉 average internal energy of CYP-lanosterol complex. Calculations of total energy of the complex's between the CYP's and POR showed that the wild type protein in complex with POR had the most favorable total energy while the others had significantly less favorable calculated total energies (Figure 2A). Especially, mutant D152G has almost zero binding enthalpy between CYP51A1 and POR. Calculations of binding energy between lanosterol in the various CYP proteins had shown that on average lanosterol is most favorably bound in the wild type, followed by the R277L mutant, while in the R431H and D152G mutants lanosterol is bound significantly less to the interacting molecules (Figures 2B,C). This was shown for calculations in aqueous solution and in complex with POR. Figure 3 show interactions between CYP51A1 (wild type and three mutants) and POR amino acid residues including FMN and heme.

### CO-difference spectra of mutants

**Figure 4:**
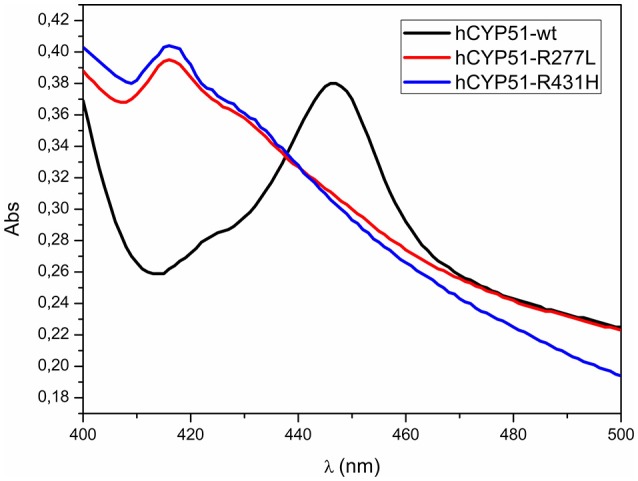
CO-difference spectra for wild type hCYP51-wt (black line), hCYP51-R277L (red line), and hCYP51-R431H (blue line) protein.

We prepared two mutant CYP51A1 proteins R277L and R431H (Table 3). We used site directed mutagenesis and mutated a *CYP51A1* insert in expression vector. Expression level determined from absolute spectra (420 nm) was 2 times reduced for mutant R277L and 3 times for mutant R431H. Western blot analyses using CYP51A1 specific antibody confirmed the presence of CYP51A1 protein in the extracts (Supplementary Figure 1). Wild type hCYP51A1 spectrum shows a typical CO-difference spectrum, with a peak at 417 nm representing catalytically inactive form of hCYP51 and a peak at 447 nm representing the active form of wild type hCYP51A1 as a result of coordinated CO in the active site (Figure 4). Mutants with amino acid changes R431H and R277L were unable to produce a peak at 447 nm and were therefore inactive. The preparation of the hCYP51A1 mutant protein was performed several times and in two different laboratories always yielding a protein with no P450 spectrum.

## Discussion

CYP51A1 is an essential housekeeping enzyme with only few known associations with diseases in humans. We searched human natural *CYP51A1* SNPs to find the damaging variants, performed molecular dynamic modeling for wild type, three selected variants, and measured CO-difference spectra of proteins. Reported damaging variants are rare variants often reported only once, which is in agreement with the requirement of this protein for normal embryonal development and adult life. In addition, only few variants have been connected to any phenotype in humans. This indicates that damaging missense variants are highly unfavorable and have not been preserved in the human populations. Clinically, *CYP51A1* variants have been proposed to be included in genetic testing in certain clinical conditions, such as pediatric cataract, to enable a more accurate genetic counseling (Gillespie et al., 2014). On the other hand, *Cyp51a1*^+^*/*^−^ heterozygous mice indicate that this genotype renders subjects potentially more susceptible to detrimental effects of unhealthy life style. Therefore, we propose to include selected *CYP51A1* variants into personalized diagnostics panel for evaluating risks for pediatric cataract, neonatal hepatic failure, global developmental delay, azole susceptibility, and cardiovascular and metabolic diseases.

We selected three mutants with predicted deleterious effects on protein activity, prepared mutant proteins *in vitro*, and compared them with wild type protein using molecular dynamic simulations. Aspartate at position 152 lies within SRS1 region and is part of a highly conserved CYP51A1 signature present among all kingdoms (Lepesheva et al., 2003). Exception are *T. brucei* and *L. major* where alanine is at this position. *In vitro* mutants of aspartate to alanine had no effect in rat Cyp51a1, but significantly decreased activity of human CYP51A1 (Nitahara et al., 2001; Lepesheva et al., 2003). In human SNP database, two substitutions of aspartic acid are reported, to glycine and to asparagine. Both are predicted to be pathogenic and we selected D152G mutant to test the effect of this mutation using molecular modeling. Results indicate that this amino acid change dramatically affected different CYP51A1 interactions with its substrate lanosterol and obligatory redox partner POR. Therefore, we can propose that this variant is highly pathogenic and results in a less active or even in inactive protein.

Interestingly, among SNPs associated with any phenotype in humans almost all are outside parts of the CYP51A1 known to be critical for enzymatic activity. Exception is leucine change to proline at position 232 lying within SRS2. Missense variants associated with any phenotype in humans are all rare variants and are predicted to be damaging by Polyphen-2 and SIFT. One of these variants is termination of protein at 421, which very likely results in no active protein. Another rare variant associated with a mild phenotype is R277C (Aldahmesh et al., 2012), which is at the end of helix G and changes a large positively charged arginine to smaller uncharged but polar cysteine. We selected a similar variant where large positively charged arginine is changed to small non-polar leucine (R277L). We were not successful in *in vitro* preparation of this mutant, but molecular modeling gave us some hints about the potential effect of this variant. Calculations of binding enthalpies indicated that this variant has lower binding properties toward redox partner POR compared to the wild type; however, binding of lanosterol is not affected. Therefore, we could predict that poorer interactions with POR would indicate that R277L variant would have a potentially lower enzymatic activity. Since the change to cysteine has been shown to be associated with pediatric cataracts, we can confirm that protein activity is affected also *in vivo* in humans.

Another mutant we selected to test was arginine change to histidine at position 431. This variant lies in a region with unknown function; however, it is highly conserved among CYP51A1 members from all kingdoms. Again, we were not successful in preparing *in vitro* the mutant protein; however, molecular dynamic modeling gave us some hints about the nature if this variant. R431H variant has unfavorable interactions with lanosterol but the variant does not affect interactions with POR. We could speculate that this variant could have potentially lower enzymatic activity but additional evidence to support this conclusion would be required.

It is not known how human CYP51A1 actually interacts with POR, therefore, we predicted amino acid residues (K127, A172, K175, K364, K442, R452, N459) to be involved in interaction with POR using CYP2D6 and CYP2B4 as a model. Only three rare variants exist at these positions. One is at position 172 where an alanine is changed to valine, both are nonpolar but valine is larger the alanine. However, both Polyphen-2 and SIFT predicted this variant as benign/tolerated. Two variants are at position 452 where arginine is changed to cysteine or histidine. These two variants are predicted by Polphen-2 as benign but deleterious by SIFT. Additional data would be needed to fully evaluate the consequences of these variants on interaction with POR.

Other interesting variants are at the positions predicted to be involved in azole interactions with CYP51A1 (Strushkevich et al., 2010). As large tryptophan at position 245 and 250 is changed to smaller serine this could affect the interaction with azoles, affecting individual susceptibility to azole treatments. Another amino acid potentially involved in azole interaction is Y151, where hydroxyl group of tyrosine is sterically interfering with fluconazole. A mutation of human Y151 to histidine increased affinity for fluconazole (Bellamine et al., 2004). Therefore, variant Y151D could also have a different affinity for azoles, affecting individual susceptibility to azoles. Y151 is part of the CYP51A1 signature at SRS1 and a change from tyrosine to aspartic acid was predicted to be damaging (Lewinska et al., 2013). This tyrosine was shown before to form an H-bond with heme and molecular modeling predicted that the change to smaller aspartic acid would make the distance to heme too large to form an H-bond. This position was also predicted to interact with POR and a change of polar uncharged amino acid to negatively charged amino acid could affect interactions with POR.

Y137 is proposed to interact with heme and is important for enzymatic activity. Interestingly, although tyrosine can be substituted by phenylalanine, this change at position 137 resulted in no protein and activity (Lepesheva et al., 2003). This indicates that OH-group is essential for catalytic activity. A mutation of rat CYP51A1 tyrosine to serine, which has an OH-group but no aromatic group, also resulted in no expressed protein (Nitahara et al., 2001). Mutation of tyrosine to cysteine, present in human population, brings in a nonaromatic amino acid with SH group. However, according to *in vitro* mutants it seems that any amino acid change at this position is highly unfavorable and we can postulate that this mutation results in a defective protein in humans. The RRR (257–259) motif in SRS3 is proposed to interact with the membrane phosphates through salt-bridges (Strushkevich et al., 2010). Three missense mutations exist at position 258 in the middle of RRR motif. Mutation of arginine is to cysteine, leucine, or histidine. The change to leucine and histidine was predicted to be benign, and change to cysteine was predicted pathogenic. As these amino acids do not form readily salt bridges, we could predict a weakening of the interaction with the membrane. Another CYP51A1 signature is HTS region in SRS4 where only one SNP exists (Lepesheva and Waterman, 2007). This is histidine 320 replaced by proline. Mutating the histidine to phenylalanine, alanine, lysine or aspartate lowered activity of rat Cyp5a11 and replacement of histidine to aspartate or asparagine also dramatically affected activity of *M. tuberculosis* CYP51A1 (Nitahara et al., 2001). H320 is necessary for proton transfer for the formation of activated oxygen. These results indicate that at this position an aromatic amino acid is necessary, for substitution with an aliphatic side chain proline, we could expect a lower activity of such protein.

In conclusion, CYP51A1 is an essential enzyme and only rare variants affecting enzymatic activity exist in the human population. Using molecular dynamic modeling we showed that two missense variants, D152G and R277L, probably affect enzymatic activity also through lowered interaction with obligatory redox partner POR. We propose to include damaging *CYP51A1* variants in genetic testing to increase the discovery of underlying causes for diseases such as pediatric cataract neonatal hepatic failure, global developmental delay, azole susceptibility, and cardiovascular and metabolic diseases.

## Author contributions

TR and IO searched for *CYP51A1* SNPs. TR, IO, and SG prepared *in vitro* CYP51A1 mutants. MS and FM performed molecular dynamical simulations. DR, TR, SG, and FM designed the study. All authors contributed to drafting of the paper.

### Conflict of interest statement

The authors declare that the research was conducted in the absence of any commercial or financial relationships that could be construed as a potential conflict of interest.
