# Supplementary figures and images for: Evaluation of Selected CYP51A1 Polymorphisms in View of Interactions with Substrate and Redox Partner
Source: Front Pharmacol. 2017 Jun 30;8:417. doi: 10.3389/fphar.2017.00417 (PMC5492350; doi:10.3389/fphar.2017.00417)

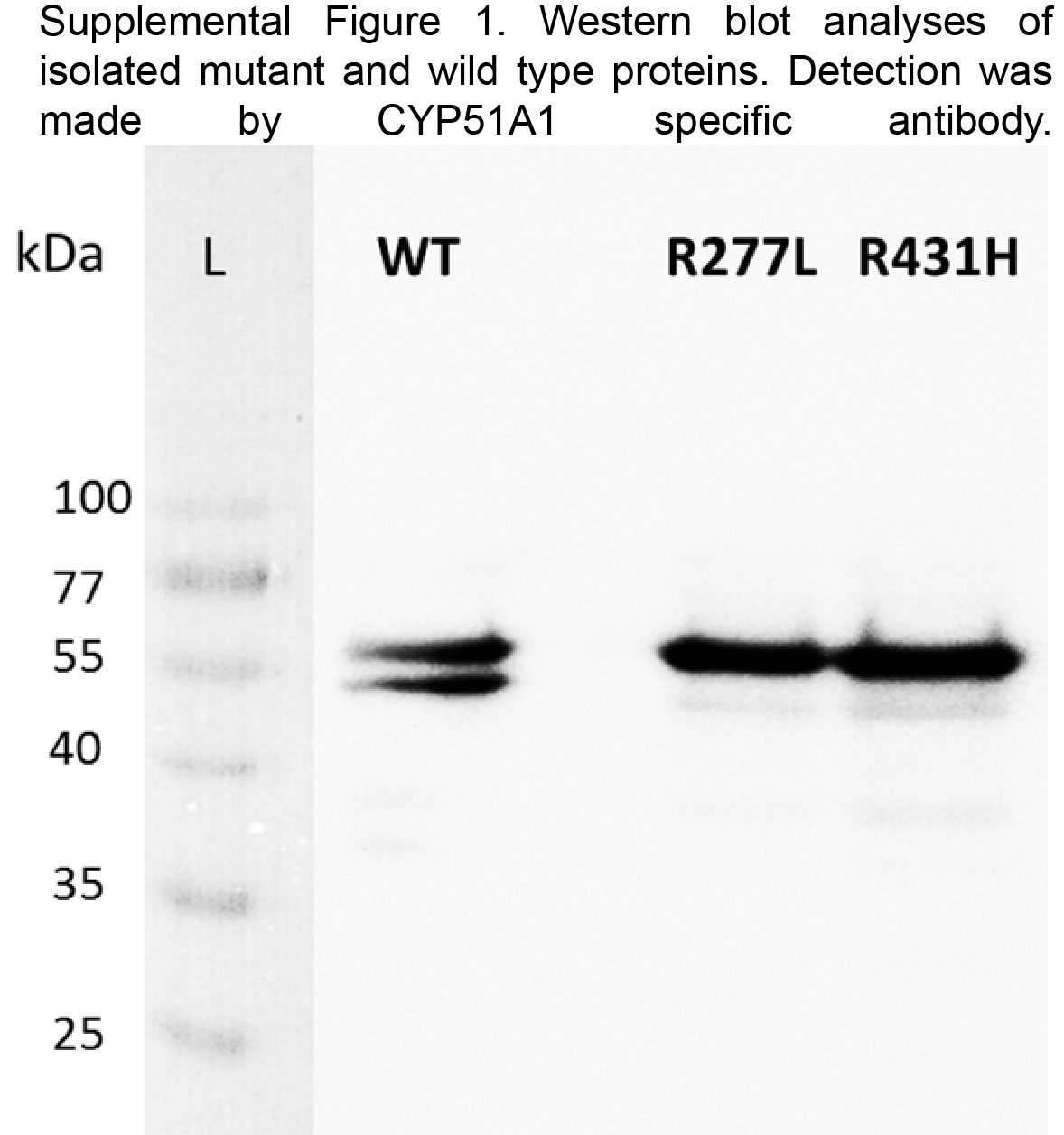

Supplement: Supplementary file 1 [file Image1.jpg]
